# Evaluation of a Laboratory-Scale Gas-Atomized AlSi10Mg Powder and a Commercial-Grade Counterpart for Laser Powder Bed Fusion Processing

**DOI:** 10.3390/ma15217565

**Published:** 2022-10-28

**Authors:** Fabrizio Marinucci, Alberta Aversa, Diego Manfredi, Mariangela Lombardi, Paolo Fino

**Affiliations:** 1 Department of Applied Science and Technology, Politecnico di Torino, Corso Duca degli Abruzzi 24, 10129 Turin, Italy; 2 Center for Sustainable Future Technologies CSFT@Polito, Istituto Italiano di Tecnologia, Via Livorno 60, 10144 Turin, Italy; 3 Consorzio Interuniversitario Nazionale per la Scienza e Tecnologia dei Materiali (INSTM), Via G. Giusti 9, 50121 Firenze, Italy

**Keywords:** powder, gas atomization, AlSi10Mg, flowability, morphology, particles size distribution

## Abstract

Laser powder bed fusion (LPBF) is an additive manufacturing technology that implies using metal powder as a raw material. The powders suitable for this kind of technology must respect some specific characteristics. Controlled gas atomization and post-processing operations can strongly affect the final properties of the powders, and, as a consequence, the characteristics of the bulk components. In fact, a complete characterization of the powders is mandatory to fully determine their properties. Beyond the most used tests, such as the volume particle size distribution (PSD) and flowability, the PSD number, the Hausner ratio and the oxidation level can give additional information otherwise not detectable. The present work concerns the complete characterization of two AlSi10Mg powders: a commercial-grade gas atomized powder and a laboratory-scale gas atomized counterpart. The laboratory-scale gas atomization allows to better manage the amount of the fine particles and the oxidation level. As a consequence, a higher particle packing can be reached with an increase in the final density and tensile strength of the LPBF bulk samples.

## 1. Introduction

Additive manufacturing (AM) technologies are rapidly spreading in several industrial fields. Therefore, the request for metallic powder with specific characteristics for techniques, such as laser powder bed fusion (LPBF), is constantly increasing. 

Atomization is undoubtedly the leading technology used to obtain metallic powder suitable for AM processes. The most established commercial atomization methods are water atomization, plasma atomization and gas atomization. With water atomization, it is possible to obtain irregular particles with a high production rate at a low cost [1]. With plasma atomization, perfectly spherical particles can be produced with a high yield of fine particles, suitable for LPBF technology [2]. Notwithstanding this, plasma atomization presents high costs and probably for this reason the sector of AM metallic powder production is still led by gas atomization [3]. This technology allows the production of spherical particles with a good yield of fine particles, with a lower cost than plasma atomization and the advantage of requiring raw materials in an ingot form [1]. A gas atomizer consists of a furnace for melting the raw material under a protective atmosphere in an atomization chamber. Here, a thin flow of melted liquid is introduced and then dispersed into small droplets under the high pressure of inert gas. During the fall, the droplets can solidify, becoming spherical particles [3]. On the basis of the atomization parameters (i.e., viscosity of the melted metal and its flow rate [4], superheating temperature [5], gas pressure [6] and nozzle diameter [7]), it is possible to control the powder characteristics, such as the particle size distribution (PSD), the impurities content, the satellites and irregularly shaped particles presence. 

To define whether a metallic powder is suitable for AM processes, the standard ASTM F3049-14 can be considered [8]. This standard summarizes the main features to consider before processing a new powder: PSD, particle morphology, rheological behavior (flowability, tap density and apparent density) and oxygen content. These analyses can give an idea of the general behavior of the powders and generally, in the literature, it has been demonstrated that they are useful to correlate the powder properties to the AM processability of the powder [8].

Mussatto et al., for example, performed powder characterization analyses on 316L powders produced by different suppliers. Their analysis included PSD, morphology and flowability, with the purpose of understanding which supplier was the best choice, in terms of powder quality [9].

Generally, in powder characterization, the first feature to evaluate is the PSD, which significantly influences the final components’ quality [10]. For example, Averardi et al. asserted that the PSD has a substantial impact on the powder layer packing density, in correlation with the layer thickness and the geometric resolution of the components. In particular, they stated that, particles smaller than 10 µm tend to agglomerate, negatively affecting the flowability [11]. Hannah et al. studied the influence of the finer particles on the properties of the powders. In their work, they mixed a batch of coarse particles of 316L with finer ones. They found that the mixed powder presented poor flow characteristics (Hausner ratio), with respect to the initial batch of large particles [12]. Another important contribution of the PSD, is in the laser absorption coefficient. Boley et al. asserted that the size distribution of the powder and their geometrical arrangement are essential factors to control for LPBF process optimization. In particular, in their study, they found that a bimodal PSD can drastically increase the absorptivity of the material, with respect to a monomodal one. This result is more evident in materials with a high reflectivity to the laser [13]. 

Both the PSD and the particle morphology, together with many other factors, have a substantial effect on the powder flowability, which significantly influences the LPBF processability. Engeli et al., for example, characterized several batches of IN738LC powder, to understand the influence of powder properties on their processability. They found that the powder with an insufficient flow behavior and a low apparent density presented some issues during the recoating phase, which negatively affected the densification of the part [14].

Finally, a significant feature of the powder that directly influences the AM parts consolidation and properties, is its chemical composition and, in particular, the impurities content (e.g., oxygen). Leung et al. demonstrated that the powder oxide content could be related to the handling and inadequate storage conditions. The presence of an oxide layer surrounding the particles, can alter the thermodynamics of the melt pool formation, since it can behave as a nucleation site for the pores, negatively affecting the densification of the components [15].

Following these considerations, it is clear that a complete characterization is mandatory to fully determine the powders’ properties. The AM literature covers the powder characterization and the influence on the final components’ properties, making comparisons between the different powder batches [5,10], but also between the different powder production techniques [16,17]. However, most of the powders characterized in the literature, are produced by different industrial suppliers that work with large-scale atomization plants, used for massive production. Moreover, several studies were conducted on AlSi10Mg powders produced with laboratory-scale gas atomizers, but these studies focused on the atomization process [15,16,17,18]. Furthermore, a recent study described the advantages, in terms of the control and stability of the atomization conditions achievable with a laboratory-scale gas atomizer [19]. In an another study, it was highlighted that the powder produced with a laboratory-scale atomizer fulfilled all of the necessary requirements for the AM processes. In addition, it was asserted that, in the laboratory-scale setup, the oxygen control of the powder can be further improved thanks to the freedom of choice of the higher purity raw materials and processed gas [20]. Despite this, to the best of the authors’ knowledge, no work, on the comparison between a laboratory-scale produced powder and a commercial one, has yet been published. 

Otherwise, this work focuses on the comparison between the commercial-grade and a laboratory-scale AlSi10Mg gas atomized powders. In particular, the two powders that were obtained through the same process but at different scales, were characterized to underline the differences in their properties and in the characteristics of the bulk LPBF samples.

Using a laboratory-scale gas atomizer allows a better control of the process and the successive post-processing steps, compared to an industrial-scale production. This can significantly affect the powders characteristics and, as a consequence, the final properties of the bulk samples.

## 2. Materials and Methods

### 2.1. Materials

The powders used in this work were a commercial-grade gas atomized AlSi10Mg, supplied by Concept Laser (commercial name CL31, Lichtenfels, Germany) and a laboratory-scale gas atomized AlSi10Mg, defined as homemade (HM). A HERMIGA 100/10 VI gas atomizer by PSI was used to produce the HM powder. The melting temperature during the atomization was around 800 °C, the atomization pressure was 40 bar, and the top pressure (pressure applied in the melting chamber) was 0.25 bar. The diameter of the nozzle for the atomization was 2.5 mm, and the material of the crucible used for melting was alumina. The composition of the CL31 powder and the ingot used to produce the HM powder is reported in Table 1.

Thanks to the atomization parameters used, after the production the atomization yield resulted at 78%, calculated as the ratio between the powder weight and the initial weight of the ingot. The HM powder was then sieved in the range of 20–50 µm, obtaining a fraction of about 30% of the total powder amount.

Moreover, the CL31 powder was used in the “as-received” state, in order to exploit it as reference powder for the LPBF system used in this work.

### 2.2. Methods

For both powders, according to the standard ASTM F3049-14 [4], the following characterizations were carried out:

- The volume PSD analysis was performed with a Mastersizer 3000 Malvern Panalytical, (Malvern Instruments, Malvern, UK).
- The morphology of the powders was observed by a scanning electron microscope (SEM) using a Phenom Pro X (ThermoFisher, Waltham, MA, USA).
- For the number PSD detection, the SEM images were analyzed with ImageJ (version 1.52t, https://imagej.nih.gov/ij/download.html, accessed on 1 August 2022), an image processing software in Java, developed by the National Institutes of Health, United States (The images analyzed per samples were about 130, with a range of 50,000–100,000 particles analyzed per sample.
- The flowability and tap density tests were performed with instruments compliant with the standard ASTM B213 and ASTM B527-22 [21,22]. In particular, the flowability was carried out on three samples of 50 g per powder. A cylinder of 100 mL with 100 g per powder was used to perform the tap density. The test was repeated three times.
- The oxygen (O), nitrogen (N) and hydrogen (H) levels of the powders were detected with a ONH836 analyzer, by LECO (LECO, St. Joseph, MI, USA). Three samples of 0.2 g per powder were analyzed, and the result was an average of these measurements. LECO supplied the program used for the heating cycle and testing, which is compatible with aluminum alloys.

Cubic bulk samples 10 × 10 × 10 mm^3^ were produced using a Concept Laser Mlab cusing R. It is a LPBF machine with a 9 × 9 cm^2^ platform equipped with a fiber laser with 100 W maximum power, a 1070 nm wavelength and a laser spot size of 50 μm. The process parameters used are the default programs for AlSi10Mg powders, provided by Concept Laser. The samples were separated from the building platform through wire electrical discharge machining. Then, the samples were cut along the building direction and polished to silica (0.025 µm). A LEICA DMI 5000 M optical microscope (Leica, Wetzlar, Germany) was used for the porosity analysis and to take ten images per sample. The porosity was evaluated by image analysis using ImageJ. Microstructural investigations at a high magnification were obtained using a field emission scanning electron microscope (FESEM) ZEISS SUPRA TM 40 (Oberkochen, Germany). For the FESEM analyses, the samples required chemical etching using the Keller solution for 10 s. The hardness tests on the cubic samples were performed using a DHV-1000 digital micro-Vickers durometer (Scope Instrument Co., Chongqing, China). Ten tests per sample were performed on the XZ cross-section. In order to perform the tensile strength test, the flat samples were produced in the XY direction (parallel to the building platform) and tested with a Zwick–Roell Z050 tensile tester (Ulm, Germany). The samples were produced with dimensions compliant with the standard ASTM E8/E8M [23], with a thickness of 3.5 mm and a width of 6 mm (sub-size specimen). Three samples were produced with each powder and tested. 

## 3. Results and Discussion

### 3.1. Powder Characterization

**Figure 1:**
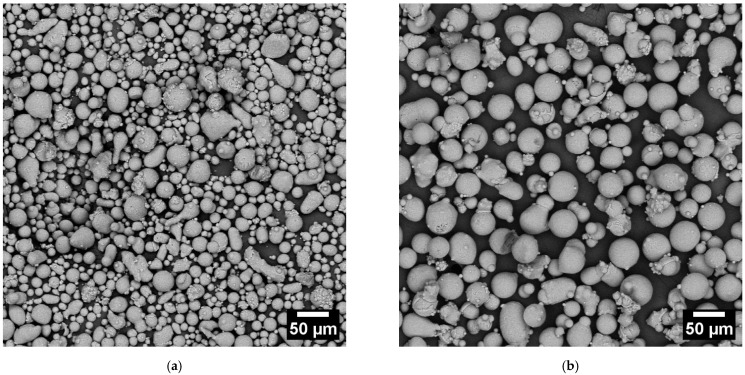
SEM images of the CL31 (**a**) and HM (**b**) powders. The magnification chosen was 500x.

The CL31 and HM powders were characterized in terms of morphology through SEM analyses (Figure 1). Most particles of the gas atomized powders are spherical. Notwithstanding this, some agglomerates and very fine particles were present. The strong interaction force causes the agglomerates between the fine particles, which tend to stick to each other creating irregular powder clusters [11,16]. In addition, some large and irregular particles were present and detected in both cases. 

**Figure 2:**
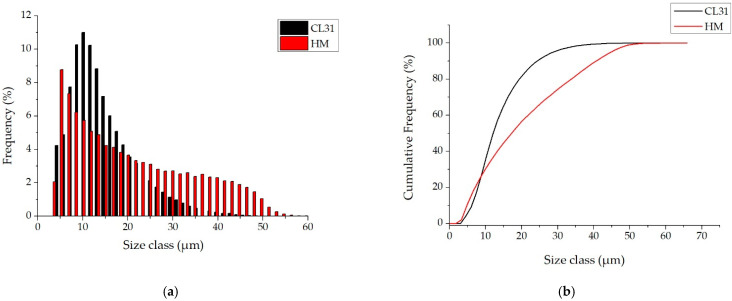
PSDs number of the CL31 and HM powders. (**a**) relative PSD frequency and (**b**) cumulative PSD frequency.

In order to deeply analyze the granulometry of the powders, image analyses were performed on the SEM micrographs, similar to those of Figure 1, and the PSDs number (Figure 2) were obtained.

This kind of analysis gives information about the number of particles measured at each diameter [24]. Considering this, the CL31 and HM powders presented very different PSD number. In fact, as it can be noticed from the PSD number graphs in Figure 2a, the majority of the CL31 particles are in the range of 5–30 µm. The HM showed a wider distribution with particle diameters between 5 and 50 µm. In addition, the CL31 powder is characterized by a high number of finer particles (with a mean diameter around 10 µm), whereas in the case of the HM, a higher number of coarser particles (with diameters between 30 and 50 µm) were detected.

To better underline the differences, in terms of dimensions, the cumulative curves (Figure 2b) are considered, to calculate the $D_{10}$, $D_{50}$ and $D_{90}$ values. The results are reported in Table 2.

In the first part of the cumulative curves, between 0 and 10 µm, the trends of the CL31 and HM powders are similar. Then, the curves present a significant difference, the HM particles are coarser than the CL31 ones. This result is evident also from the difference in the $D_{50}$ values, and even more, in the $D_{90}$ values.

**Figure 3:**
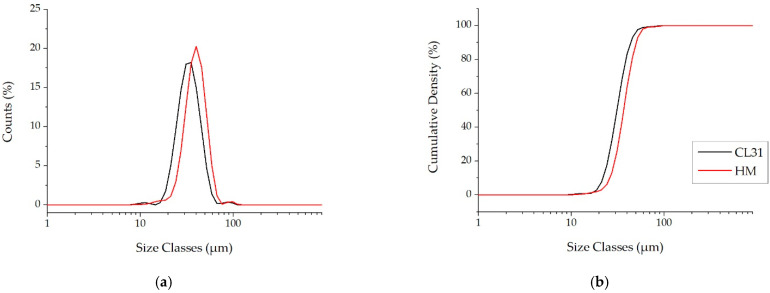
PSD Volume of the CL31 and HM powders. (**a**) relative PSD frequency and (**b**) cumulative PSD frequency.

Moreover, as generally used for the AM powders, the PSD volume analyses were also carried out. This kind of analysis is a measure of the space occupied by the particles, with a specific size. For this reason, the presence of finer particles is highlighted less effectively [24]. In fact, contrary to the PSD number, the PSD volume of the CL31 and HM (Figure 3a) are similar. In both cases, in fact, a monomodal distribution was obtained. In particular, just a slight shift to lower diameters was recorded in the CL31 distribution, with respect to the HM one. The values of $D_{10}$, $D_{50}$ and $D_{90}$, related to the cumulative curves shown in Figure 3b, are reported in Table 3. In this case, slight differences were detected in all D values. 

In agreement with the standard F3049-14 [8], in order to understand the processability of the powders, in terms of rheology, different analyses were performed. The first test was the flow rate. Both batches showed a no flow behavior in both funnel types: Hall and Carney (Table 4). Then, the apparent and tap densities were measured and the obtained values are reported in Table 4. As concerning the former, similar values were obtained, whereas in the case of the latter a higher value was recorded for the CL31 powder, suggesting a less efficient packing. 

Finally, the Hausner ratio of the powders was calculated (Table 4), as the ratio of the tap and apparent densities. According to Sutton et al., when a Hausner ratio below 1.25 is obtained, the powder can be considered free-flowing [25]. For both batches, the value of the Hausner ratio was below 1.25, but the CL31 value was higher than the HM value. The CL31 powder presented a Hausner ratio value similar to that of a gas atomized commercial AlSi10Mg powder, described by Haferkamp et al. [26]. Sutton et al. stated that the evaluation of the powder flowability through the Hausner ratio or the flowmeters (Hall and Carney) often results in an overgeneralization [25]. A “no flow” behavior in the flowmeter does not necessarily mean a high value of the Hausner ratio, or vice-versa, since the powder flowability is affected by different factors that are not fully detectable with these techniques. In fact, both analyzed powders presented a “no flow” behavior, even if their Hausner ratios are different. Abdullah et al. demonstrated that the Hausner ratio is influenced by the friction between particles that depends on the particle shape, surface oxides and other factors [27]. According to the literature, a low Hausner ratio denotes a high powder packing [26] that is also influenced by a limited amount of fine particles [17]. 

Taking into account the role of the oxidation phenomena in friction between particles and consequently in Hausner ratio, the ONH analyses were performed for both powders. As shown in Table 5, the O, N and H contents of the CL31 powder are higher than in the HM powder. 

Aluminum powders always present an oxide layer on the surface, formed during a specific step of the atomization process, which is passivation. The whole gas atomization process is conducted in a controlled atmosphere. The melting phase is performed under vacuum, and the gas used during the atomization phase is inert (Ar). Consequently, if there are no leakages or critical issues during the process, the powder remains in an oxygen-free environment while being produced. The reaction of the powders in contact with oxygen is highly exothermic. The explosibility of the aluminum powders increases, in fact, with the decrease of its dimensions [28]. For this reason, a passivation step is needed before collecting the powders. This phase has to be carefully controlled, gradually exposing the powder to the air, before safely handling it.

The oxidation level of the powders could derive, in the first place, from the passivation conditions, but also from successive operations. As confirmed by Leung et al., the different content of O and N in the powder could derive from the successive handling operations, transportation and storage conditions [15]. The lower levels of O, N and H in the HM powder suggest a more controlled atomization and handling operations, due to the laboratory-scale process. 

### 3.2. Bulk Samples Comparison

Following the analyses, both powders were used to produce bulk LPBF samples, in order to compare the final properties of the components. 

**Figure 4:**
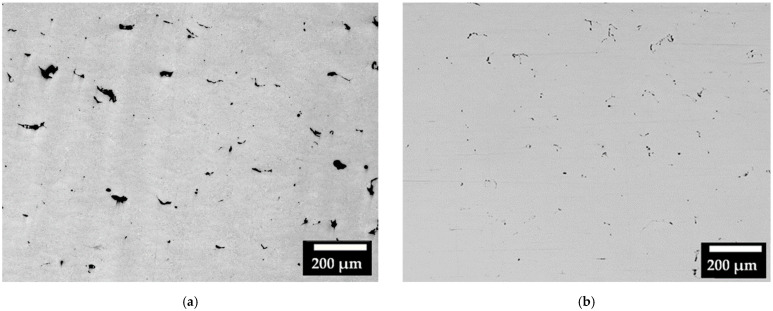
Micrographs of (**a**) CL31 and (**b**) HM, XZ cross-sections.

Regarding the consolidation of the samples, Figure 4 represents the micrographs of some areas (including different melted layers) observed on the XZ cross-section of the CL31 (Figure 4a) and HM (Figure 4b) samples. The images were selected as the most representative of the samples. Considering all the pictures analyzed, the relative density achieved for CL31 was 98.87 ± 0.15%, while for HM, a mean value of 99.17 ± 0.30% was obtained. In the CL31 samples, distributed irregular pores were observed, due to the poor packing density. In fact, having a high packing density allows to create powder beds with less initial voids, which lead to a higher final densification [11]. The HM samples presented a slightly higher density than the CL31 samples, probably because of the higher powder packing (Table 4), due to the more limited amount of fine particles (as visible in Figure 2) [17].

**Figure 5:**
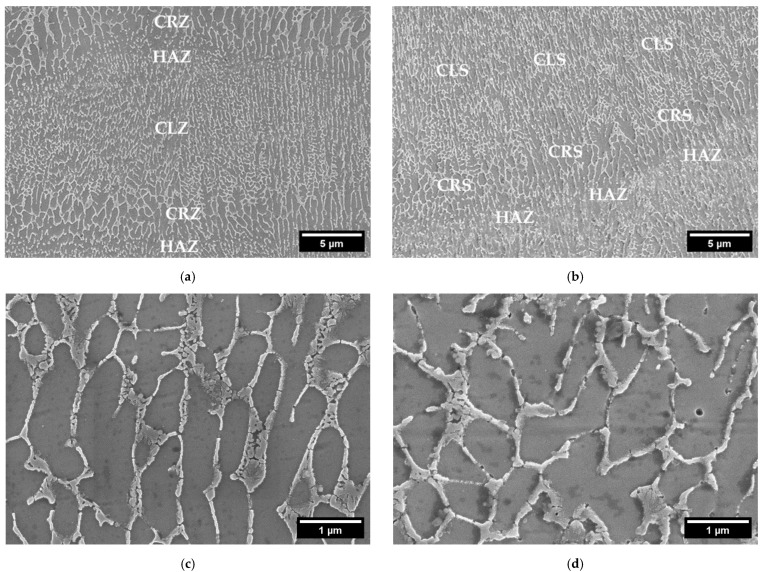
FESEM microstructures at a high magnification of CL31 (**a**) and HM (**b**) at 10 kX and CL31 (**c**) and HM (**d**) at 50 kX.

The details of the microstructure of the samples obtained with both powders, are shown in Figure 5. Here the typical microstructure of AlSi10Mg processed by LPBF, with the distinction between the fibrous Si eutectic structure and primary Al cells, is noticeable. Si forms a cellular dendritic structure, which appears columnar along the building direction, following the thermal gradient, as reported in the literature [29]. The Si network interruption takes place in the lower part of the melt pools, in the so-called heat-affected zone (HAZ). Here, the silicon diffusion rate is increased, due to the maximum heat causing a coarsening of the silicon phase and the interruption of the cellular network [30]. Moving toward the pool center, a coarse cell zone (CRZ) and finally the columnar zone (CLZ) can be detected. Considering the cell dimensions, a measurement through an image analysis was performed. An average cell width of 240 ± 30 nm was measured for CL31, against 270 ± 50 nm for HM. No significant differences were detected.

The tensile properties of the samples produced with both powders are reported in Table 6. Here, beyond the values of the tensile properties of the CL31 and HM samples, a Δ for each property was defined as the difference between the HM mean value and the corresponding CL31 mean value, expressed as a percentage, with respect to the CL31 value. It can be noticed that Δ is always a positive value, indicating that the properties of the HM samples were, in any case, higher than the CL31 samples. An interesting result was achieved regarding the yield strength (YS), since a Δ of 13% was recorded. In the case of the Young modulus (E) and the ultimate tensile strength (UTS), no significant differences were detected. Regarding the elongation at the break ($\varepsilon_{\mathrm{break}}$), even if the HM samples showed a value of 14% higher than the CL31 values, the standard deviation values were quite high, so the difference is not statistically relevant. Finally, the hardness difference between HM and CL31 was not significant.

These results are comparable with those obtained by Kempen et al., where the AlSi10Mg samples were also produced along the XY direction. The last column in Table 6 reports the complete values. The tensile test results presented a very similar value of E, but a slightly lower UTS value and a slightly higher $\varepsilon_{\mathrm{break}}$ value, with respect to the CL31 and HM samples [31].

**Figure 6:**
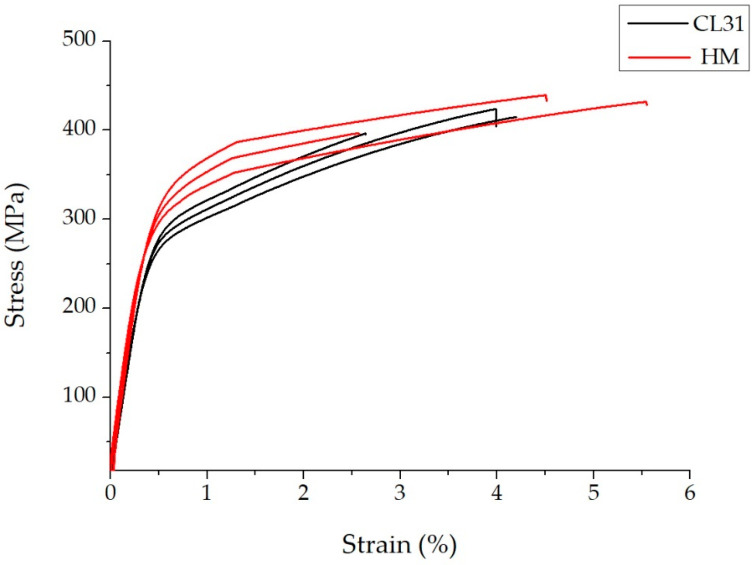
Representative stress strain curves of the CL31 and HM samples.

In Figure 6, the representative stress-strain curves of the CL31 and HM samples are presented. It is evident that the mechanical behavior of the CL31 and HM samples differs mainly for the YS values.

The higher YS value of the HM samples could be explained by a superior densification of the alloy. In fact, the HM powder presented a lower presence of fine particles and a lower oxidation level, thanks to the laboratory-scale production. For this reason, it is reasonable to suppose that the HM powder had a higher powder packing and was able to better densify during the LPBF process with the fixed parameters. In this way, the samples with a higher density and YS were obtained.

## 4. Conclusions

This work aimed to analyze two powders of the same composition but with different origins: a commercial-grade gas atomized AlSi10Mg (CL31) in its “as-received” state and a laboratory-scale gas atomized counterpart (HM), after sieving.

For both powders, the main characteristics of a powder useful for AM, were measured (following the standard ASTM F3096-14) and the bulk samples were produced via LPBF.

The main differences between the CL31 and HM powders resulted in the PSD number and oxidation level. In fact, the HM powder presented a significantly lower amount of finer particles with a lower oxidation level. These results led to better powder packing, due to less friction between the particles, which is improved by a low amount of fine particles and a low level of surface oxidation. These improvements derived from the used gas atomization process parameters, the controlled sieving step and handling operations. As a consequence, better powder packing led to a higher level of densification of the bulk samples during the LPBF process with an improvement in their yield strength.

Considering that a powder with a smaller amount of finer particles is less sensitive to a layer thickness variation [17], using the HM powder would allow the increase in the LPBF production rate by increasing the layer thickness and consequently decreasing the building time of the components.

## Figures and Tables

**Table 1:** AlSi10Mg nominal chemical composition.

Element (%)	Si	Fe	Cu	Mn	Mg	Ti	Al
AlSi10Mg	9–11	≤0.55	≤0.05	≤0.45	≤0.2–0.45	≤0.15	Balance

**Table 2:** Particle size percentile values of the powders, derived from the number cumulative curves.

Powder	$D_{10}$ (µm)	$D_{50}$ (µm)	$D_{90}$ (µm)
CL31	6.1	12.2	24.2
HM	4.8	17.1	40.7

**Table 3:** Particle size percentile values of the powders, derived from the cumulative volume curves.

Powder	$D_{10}$ (µm)	$D_{50}$ (µm)	$D_{90}$ (µm)
CL31	24.8	35.3	49.8
HM	29.5	41.8	57

**Table 4:** Flowability values of the powders.

Powder	Flow Rate	Apparent Density (g/cm^3^)	Tap Density (g/cm^3^)	Hausner Ratio
CL31	No flow	1.45 ± 0.02	1.65 ± 0.01	1.14
HM	No flow	1.41 ± 0.03	1.53 ± 0.02	1.09

**Table 5:** Results of the ONH analysis of the powders.

Powder	O (%)	N (%)	H (ppm)
CL31	0.0878 ± 0.0159	0.0095 ± 0.0005	54.9 ± 0.9
HM	0.0287 ± 0.0007	0.0048 ± 0.0002	20.1 ± 22.7

**Table 6:** Mechanical properties of the CL31 and HM samples, compared with the literature.

Powder	CL31	HM	Δ (%)	AlSi10Mg [31]
E (GPa)	65 ± 4	65 ± 4	0	68 ± 4
YS (MPa)	286 ± 8	323 ± 14	13	-
UTS (MPa)	412 ± 14	422 ± 23	2.4	391 ± 6
$\varepsilon_{\mathrm{break}}$ (%)	3.6 ± 0.8	4.1 ± 1.5	14	5.5 ± 0.4
HV	135 ± 9	139 ± 5	3	-

## Data Availability

Not applicable.
